# Research on smart city public health detection system and improvement technology based on intelligent multi-objective

**DOI:** 10.3389/fpubh.2024.1347586

**Published:** 2024-03-28

**Authors:** Bo Liu, Jie Gu, Chao Wang

**Affiliations:** Wuhan University of Science and Technology, Wuhan, China

**Keywords:** smart cities, public health, health management, detection systems, public transport, living environment

## Abstract

**Introduction:**

With the increase of urban population density, urban sanitation becomes more severe; urban sanitation has important influence on public health. Therefore, in order to realize the detection of public health in smart cities, the research will use cutting-edge scientific and technological methods to improve urban environmental health, so as to promote the realization of public health achievements. This study introduces public health detection and optimizationtechnologies for smart cities.

**Methods:**

Firstly, a data detection system for urban public health environment was established using sensors and intelligent multi-objective technology to evaluate the water quality, air quality, and noise level of the city. Then, an intelligent garbage management system based on Tensor-flow was constructed to achieve efficient garbage collection and treatment. Finally, an intelligent traffic management system was developed to monitor and regulate urban traffic flow.

**Results:**

The results of the simulation experiment demonstrated that the life data detection system was operationally stable, with a high success rate of 98%. Furthermore, its accuracy in detecting residents’ living environment data was above 95%, the maximum relative error was only 0.0465, making it a reliable and efficient tool. The waste recycling system achieved a minimum accuracy of 83.6%, the highest accuracy rate was 95.3%, making it capable of sorting and recycling urban waste effectively. Additionally, the smart traffic management system led to a 20% reduction in traffic congestion rates, 20 tonnes less tailpipe emissions and an improvement in public health and well-being.

**Discussion:**

In summary, the plan proposed in this study aims to create a more comfortable, safe, and healthy urban public health environment, while providing theoretical support for environmental health management in smart cities.

## 1 Introduction

With the rapid growth of urbanization and population, there are tremendous challenges and pressures faced by urban sanitation and public health (PH) (1, 2). Environmental pollution, traffic congestion, and waste disposal have significantly impacted the quality of life and health of residents. Therefore, the improvement of urban environmental health and upgrading of PH have become pressing tasks. Despite efforts to test residential environments, there persist major challenges. Firstly, water quality monitoring in the traditional sense necessitates water sample collection, which is then analyzed in a laboratory. However, this process is both time-consuming and laborious and does not enable changes to be monitored in real time (3). Additionally, certain pollutants may have low concentrations and could potentially go undetected by conventional detection methods. Furthermore, air quality monitoring presents an array of various complex air pollutants, requiring monitoring instruments with high precision and sensitivity for accurate detection and analysis. Furthermore, air pollution sources are extensively scattered, and the selection and positioning of monitoring sites must consider comprehensiveness and representativeness to guarantee precise monitoring of air quality across the city. The complexity and fluctuation of the noise spectrum in urban environments pose a challenge to noise monitoring, unlike other environmental factors. The discontinuous characteristics further exacerbate the difficulty. The accuracy of noise monitoring data is also impacted by environmental conditions like buildings and roads, affecting the propagation of sound. In the context of traffic congestion, manual management is the primary method of traditional traffic management. However, it lacks scientific and technological support, rendering intelligent management and optimal scheduling impossible. Consequently, it cannot meet the changing demands of urban traffic, leading to inconvenience for residents (4). Regarding the issue of urban waste disposal, the conventional disposal methods result in significant wastage of resources. Many types of waste contain materials that can be recycled, but often these resources are not effectively reused through traditional treatment methods. This leads to a waste of resources and an increased burden on the environment (5). To enhance urban sanitation and PH, it is essential to utilize advanced technology. With this goal in mind, the study has devised three improvement systems – the Living Environment Data Monitoring System (MS), the Waste Recycling System, and the Intelligent Traffic Management System. The implementation of these systems is expected to create a more comfortable, secure, and healthy living environment for the residents. The study is divided into four chapters. The first section describes the current status and main achievements of research by experts and scholars at home and abroad on urban residents’ environmental monitoring, urban traffic management, and urban waste disposal. Technical abbreviations are explained. The second section constructs three systems on Smart city environmental sanitation management and public health (SCESMPH). The study adheres to conventional academic structure, formal register, objective language, and clear, concise sentences with causal connections. Section 2 conducts simulation experiments and examines the effectiveness of the detection management system for living environment data, the management system for recycling rubbish, and the intelligent traffic management system. In Section 3, the simulation experiments of the three systems are analyzed, and the research’s shortcomings are identified.

The innovation points of this study can be mainly divided into two points. First, the experiment applies intelligent multi-target detection technology to smart city public health detection. By integrating the advanced Tensorflow framework and pattern recognition algorithms, efficient identification and analysis of multiple factors in the field of urban public health are achieved. Second, the experiment proposed a new system architecture that uses image recognition technology and the design of a garbage classification and recycling system based on the TensorFlow framework to achieve intelligent operation and optimize garbage management.

The main contributions can be divided into two points. First, the experiment built a public health detection system suitable for smart urban environments, which can monitor and analyze urban health conditions in real time; it achieved high-precision detection of three indicators of urban water quality, air quality, and noise. Second, the experiment successfully built a garbage classification and recycling system to promote intelligent management and efficient disposal of urban garbage. The effectiveness of the proposed improvement technology was verified through a series of experiments, especially the significant improvement in accuracy and processing speed.

## 2 Related works

Integrated improvement of SCESMPH is a crucial academic discipline that encompasses urban environments and PH management. Rapid urbanization and population growth are leading to significant issues in urban environments and PH, which necessitate an integrated approach to solving them. Mohapatra et al. elaborated on the concept and characteristics of smart cities, while also pointing out that implementing smart cities involves many fields, including urban planning, transportation, energy, environment, etc., which will face challenges in technology and technology integration. In addition, the implementation of smart cities also involves social challenges such as data privacy and security, social participation, digital divide, and social cognition (6). Therefore, scholars and experts worldwide are conducting extensive research to address these problems. In the context of environmental monitoring for urban dwellers, Mohapatra et al. implemented an intelligent garden system with zero water waste through the Internet of Things (IoT) technology to save water resources. This system utilizes IoT sensors and connectivity to intelligently manage and optimize water resources in gardens. The article provides a detailed description of the use of various sensors and intelligent controllers to monitor and control the water demand of plants, and to reduce waste by applying appropriate irrigation strategies (7). Zhao et al. designed an IoT environmental monitoring system based on multi-protocol fusion in order to improve the efficiency of the environmental monitoring system. The system achieved seamless transmission and processing of sensor data by fusing different transmission protocols. The system also employed adaptive sampling and data compression to improve the efficiency and reliability of data transmission. Experimental results showed that the system had good performance and feasibility in environmental monitoring (8). Fan et al. designed a portable noise time-frequency characteristic monitoring system for environmental assessment of power transformer rooms in order to reduce noise pollution in residential transformer rooms. The system consists of a sensor array and a vibration noise tester. After the field test, it was shown that the sound pressure in the power transformer room had a significant periodicity with an amplitude of 0.1–0.2 Pa. The main noise frequency was fixed at 300 Hz (9). In urban traffic management, Nambajemariya et al. proposed a new traffic management system based on existing VANET and IoV in response to the many problems with conventional traffic signals. The experimental results showed that this traffic management system can significantly reduce the average waiting time and increase the number of servicing vehicles (10). Mohamed et al. proposed an intelligent traffic management system based on IoT to build an intelligent traffic management system to alleviate traffic congestion in urban areas. The results showed that the system can significantly reduce urban traffic congestion (11). Du H et al. analyzed the reasons for the success of the city’s garbage classification and recycling system, taking the Shanghai garbage classification and treatment project as an example, in response to the increasing amount of urban solid waste (12). In the context of municipal waste treatment, Wang et al. proposed an evolutionary game model that incorporates government supervision, corporate treatment and citizen participation. Using this evolutionary game model, evolutionary and stabilization strategies for urban household waste separation and recycling governance were derived under four different scenarios. In addition, the effect of government incentive supervision on the strategy choices of each party was investigated through numerical analyses. The results showed that the key to promoting active participation of residents was to increase the incremental income from waste separation (13). Liu scholars proposed the application of artificial intelligence technology to the field of waste classification in response to the inefficiency of waste classification and recycling. The results showed that it can significantly reduced the labor cost and improve the efficiency of rubbish sorting and recycling (14). WenxuanZeng et al. designed a new type of intelligent sorting bin for the need of waste classification in today’s society, and the structure of each part of the bin was introduced and analyzed in detail. The experimental results indicated that this bin can realize the automatic classification of indoor rubbish after adjusted by the platform (15). Chengyu Hu scholars designed a bin with intelligent classification based on MSP430 chip with voice recognition for the problem of trash classification. The bin can achieve the classification and positioning of rubbish according to the voice information input by the user (16).

There are numerous factors to consider when aiming for the comprehensive enhancement of SCESMPH. Based on the research conducted by domestic and international scholars, current studies in this field lack diversity, and the practical application of their findings is limited. The study develops a living environment data management system, a waste recycling scheme, and an intelligent traffic control system. These innovations are anticipated to enhance the overall urban environment and promote residents’ well-being.

## 3 Design of a system on urban sanitation management and PH improvement

This chapter focuses on the design and improvement measures of three system functions. The first one is the environmental data detection management system, which mainly uses sensor technology and Internet technology to achieve real-time monitoring of urban water quality, air, and noise. In order to ensure the accuracy of data, Kalman filter function is introduced into the system. The second is the rubbish recycling system, which is based on the Tensorflow framework and enhances the recognition accuracy of the rubbish images by training the SSD model, and then commands the robotic arm to achieve the classification and transmission of rubbish according to the classification images. The third is the intelligent traffic management technology, the system design is more complex, with many functions, can achieve the comprehensive supervision of the city’s traffic and optimise the traffic routes.

### 3.1 Design of management system for detection of residential living environment data

**Figure 1:**
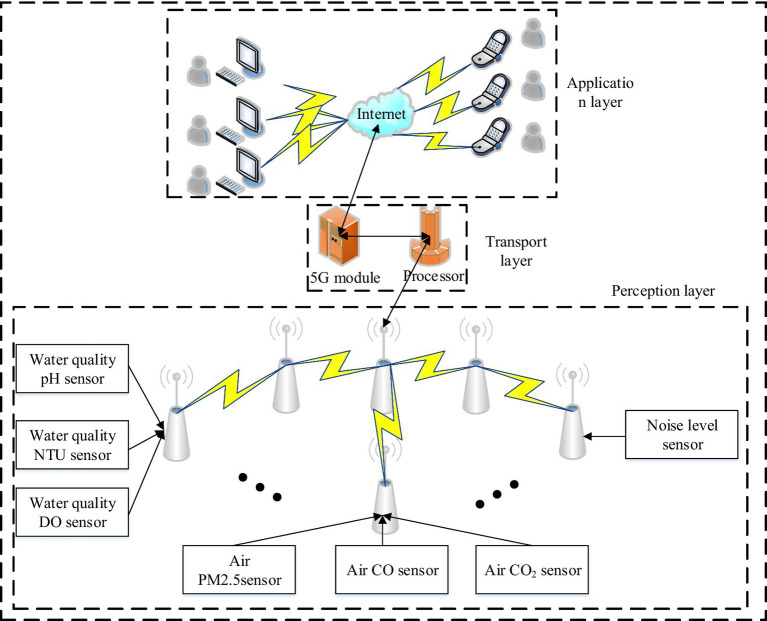
Overall design framework for MS in urban environments.

The quality of a resident’s living environment has a significant impact on people’s health and well-being. In order to ensure that the living environment of residents meets health and safety standards, it is vital to establish a reliable system for managing the detection of residential living environment data. This system will provide a timely and accurate picture of the reality of the environment by monitoring three indicators: water quality, air quality and noise levels. Based on the detection indicators of the urban environment and the functions that the system needs to fulfill, the overall framework of the wireless sensor-based environmental MS is designed as shown in Figure 1.

**Figure 2:**
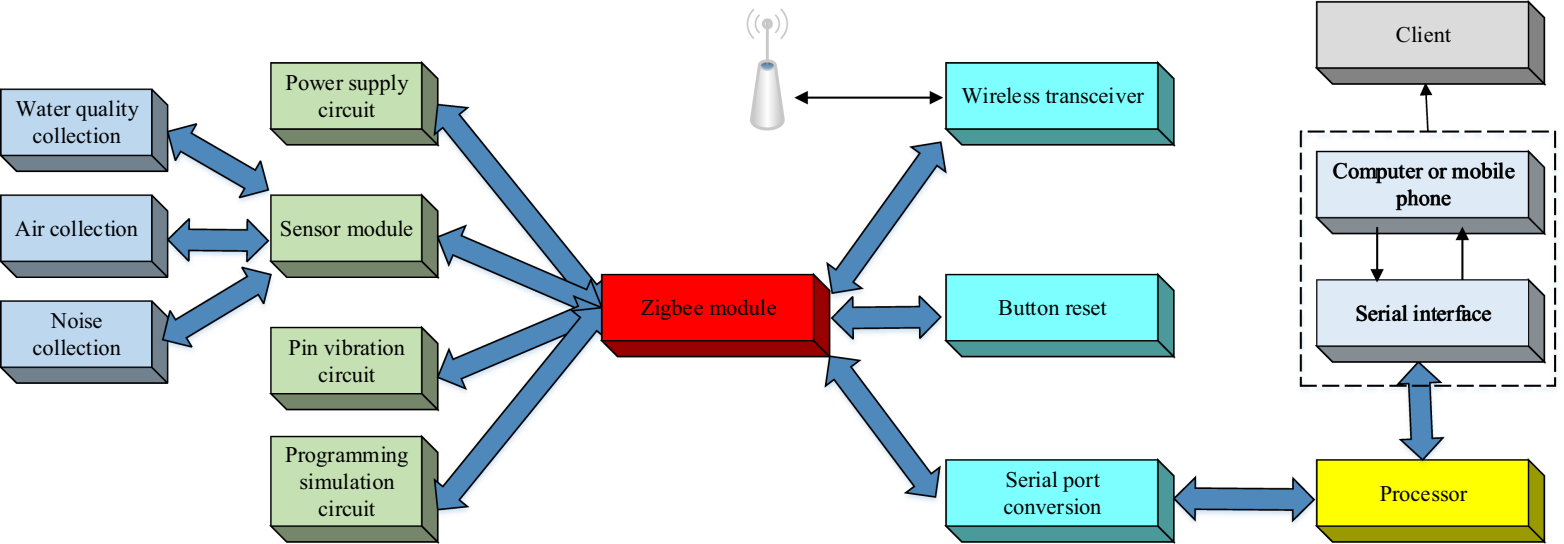
Node design schematic.

In Figure 1, this environmental MS is mainly composed of three parts: application layer, transmission layer, and perception layer. Among them, the perception layer is the sensor network, which consists of multiple sensor nodes, ZigBee terminal nodes, and a ZigBee network coordinator, and its main role is to collect and transmit water quality, air, and noise data in the urban environment. The transmission layer is mainly composed of the STM32F407 micro-controller processor, the 5G communication module, and the 5G gateway composed of the Ethernet model. The processor in this part transmits data by directly connecting to the 5G communication module or Ethernet interface module (17–19). And the data collected in the sensing layer is transmitted to the 5G gateway through the serial port, and then transmitted to the Internet under the 5G network terminal transmission protocol. And in the application layer, the main users and managers can receive the real-time detection data of the urban environment through the computer terminal and mobile phone terminal. In order to realize the system designed above, the hardware of each part needs to be designed, and the specific principle of the design of each node of the system hardware is shown in Figure 2.

The most important thing in the design of hardware system of urban environment MS is the environmental parameter sensor acquisition module. The environmental parameter sensor acquisition module is divided into water quality parameter acquisition module, air parameter acquisition module and noise parameter acquisition module. Among them, the water quality parameter acquisition module is mainly designed from the three aspects of turbidity, PH value and dissolved oxygen rate of residential water supply. For the turbidity parameter collection module, this system uses TS-2000 immersed suspended solids sensor to collect the turbidity of water quality. The sensor uses a double-beam infrared light scattering detection technology, turbidity detection of the calculation principle is shown in Eq. 1.


(1) $$NTU=K*\left(I/I_{o}\right)$$

**Figure 3:**
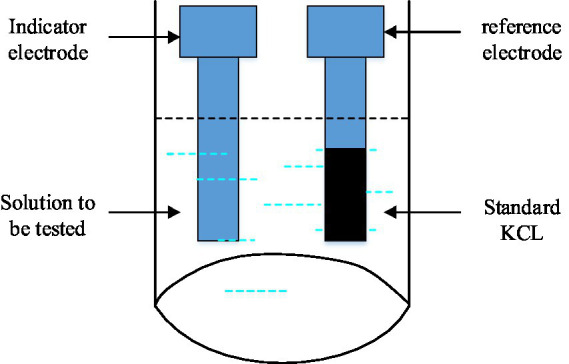
Schematic diagram of the working principle of PH sensor.

In Eq. 1, NTU is the degree of turbidity of the water; K is a constant, which can be derived from the design and final calibration of the sensor; I is the light intensity detected by the sensor; and $I_{o}$ is the reference light intensity measured in pure water. In addition, the suspension sensor uses optical band-pass filtering and modulated excitation light, which can effectively avoid the influence of ambient light and water color on the measurement data, and the built-in light source compensation measurement loop can effectively compensate for the intensity attenuation of the light source (20–23). For the PH value parameter collection module, the KNF-101-1PH glass electrode sensor is selected to collect the PH value of water quality, and the working principle of this glass electrode sensor is shown in Figure 3.

Because the PH value of water quality has correlation with the electric potential of the battery in Figure 3, the PH value of water quality can be obtained indirectly through the measurement of the electric potential, and the principle of PH value calculation is shown in Eq. 2.


(2) $$PHx-PHx=\frac{E_{x}-E_{s}}{2.30RT/F}$$

Eq. 2 where PHx is the PH value of the water to be measured; PHs is the PH value of the standard KCL solution; $E_{x}$ is the electromotive force of the solution to be measured; $E_{s}$ is the electromotive force of the KCL solution; R is the gas constant; T is the absolute temperature; CF is the Faraday constant. The glass electrode pH meter includes an electrode that is connected to the reference electrodes inside and outside the sensor. The glass electrode soaked in solution will react with hydrogen ions in water, creating a potential difference. The measuring instrument measures and converts the water quality electromotive force according to the equation in Eq. 2 (according to electrochemical theory, there is a linear relationship between the change in potential difference in the given equation and the change in pH value. In the given data, the potential difference corresponding to a change of 1 pH unit is 59.16 μV), and finally the pH value of the water quality can be seen in the upper computer. For the dissolved oxygen rate parameter collection module, the RS-LDO-N01 fluorescent dissolved oxygen sensor was selected for this system (24–26). The sensor is based on the fluorescence burst principle, where blue light irradiated onto a fluorescent substance causes the fluorescent substance to excite and emit red light. Since oxygen molecules can carry away the energy (the burst effect), the duration and intensity of the excited red light is inversely proportional to the concentration of oxygen molecules. The sensor determines the concentration of oxygen molecules by measuring the phase difference between the excited red light and the reference light. Reference light is an internal standard that is not affected by oxygen molecules. The change in phase difference is directly proportional to the change in oxygen molecule concentration. In addition, there is a calibration value inside the sensor for comparison with the measured phase difference. By comparing the measured and calibrated values, the concentration of oxygen molecules in the effluent can be calculated. The relationship between water dissolved oxygen concentration and fluorescence lifetime is shown in Eq. 3.


(3) $$I_{0}/I=\tau_{0}/\tau =1+Ksv\left[O_{2}\right]$$

In Eq. 3, $I_{0}$ and $\tau_{0}$ are the fluorescence intensity and fluorescence lifetime in the absence of oxygen, I and τ are the fluorescence intensity and fluorescence lifetime in the presence of oxygen, KSV is the bursting constant of the bursting agent, and $\left[O_{2}\right]$ is the dissolved oxygen concentration. When the dissolved oxygen data is collected by the sensor, it can be uploaded to the client through the communication module to display the dissolved oxygen concentration of water quality in real time.

The air parameter acquisition module is mainly designed from two aspects: urban air PM2.5 and CO/CO2 concentration. For the air PM2.5 parameter acquisition module, the system selects the ZPHO2 dust sensor to detect PM2.5 in urban air. The sensor heats the urban ambient air through resistance, forcing the air to rise and driving the PM2.5 in the air into the MS for detection. Firstly, the ZPHO2 dust sensor sucks air into the detection chamber inside the sensor through an integrated fan, completing the sampling of PM2.5 particles in the air. Then, when PM2.5 particles meet the laser beam in the detection room, some of the light will scatter. At this time, the ZPHO2 dust sensor will collect and measure the intensity of the scattered light. Finally, the ZPHO2 dust sensor converts the measured scattered light intensity into PM2.5 concentration. The detection principle is to use laser scattering technology to illuminate the particles in the air with the light beam emitted by the laser emitter. The particles will reflect the light intensity back to the laser scatterer, and the particle size and concentration can be calculated based on the intensity and color of the reflected light. For the detection of PM2.5, a small pore sieve needs to be installed in the ZPHO2 dust sensor. When air flows through, only particles with a diameter less than 2.5 microns are allowed to pass through, while particles larger than 2.5 microns are blocked outside the sieve. In this way, only PM2.5 particles with a diameter less than 2.5 microns pass through the sieve and are detected by the laser scatterer. By analyzing the reflected light, the concentration of PM2.5 per cubic meter of air can be calculated. For the air CO/CO2 concentration parameter acquisition module, this system uses a MiCS-4515 gas sensor. This sensor has two independent sensing elements in one package, which are used to detect the concentrations of CO and CO2, respectively. When gas enters the sensing element, it interacts with the material on the working electrode, thereby changing the conductivity of the electrode surface. This change will cause a change in the current, and the gas concentration can be determined by calculating the relationship between the measured current and the known gas concentration. For the detected signal data, analog voltage signals are used, so after the detection is completed, the analog signal is converted into a digital signal and transmitted to the terminal display to display the air CO/CO2 concentration. And the noise detection module is mainly designed from the aspect of noise level in the city. The system uses RS485 noise sensor to collect the noise in the environment, extracts the key features about the noise level after pre-processing the collected noise signal, and then calculates the noise level according to the extracted features. The noise level calculation method is shown in Eq. 4.


(4) $$L=L0+10\log10\left(I/I0\right)$$

**Figure 4:**
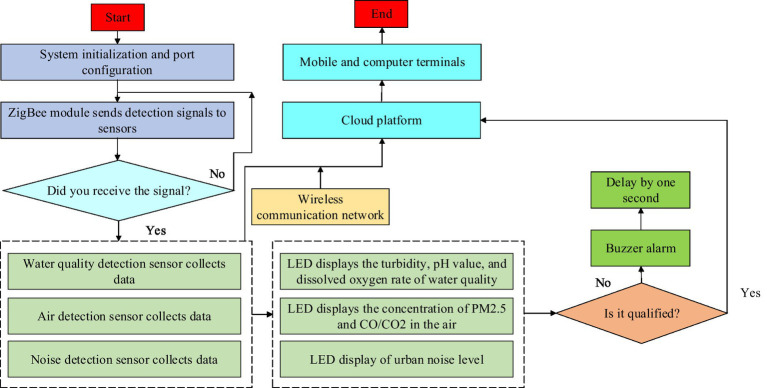
Main flow chart of the residential environmental data testing management system.

In the Eq. 4, L is the noise level; L0 is the base noise level; I is the measured sound intensity; I0 is the reference sound intensity. Finally, the noise level detection results are transmitted to the display terminal through digital signals to view the noise level in various parts of the city. Install the RS485 noise sensor in the location where it needs to be measured, such as streets, parks, etc. Connect the sensor power supply and communication lines to transmit the measurement data of the sensor to the display terminal. The sensor converts the real-time measured sound pressure signal into an electrical signal and performs analog-to-digital conversion (A/D conversion). After converting it into a digital signal, all data is sent to the data display terminal through the RS485 bus line. Analyze the transmitted digital noise data on the data display terminal using Matlab analysis software and convert it into visual noise level information. Finally, it is presented digitally on the customer’s mobile phone, computer, and other display terminals. In general, the whole process of residential living environment detection is shown in Figure 4.

However, the data collected by each sensor will have a certain error, which will affect the whole system to display the final results. Therefore, in order to improve the accuracy of the detection data, the study introduces the Kalman filter algorithm to process the detection data. The Kalman filter will use the estimated value of the current state and the state equation to predict the state value of the next time step, and calculate the corresponding covariance matrix. In the update step, the Kalman filter combines the measured values and observation equations, calculates the Kalman gain based on the measurement error and covariance matrix, and then uses the gain to update the estimated values and covariance matrix of the state variables. By repeatedly repeating the prediction and update steps, the Kalman filter can continuously improve the estimation of state variables based on previous estimates and measurement results, thereby improving the accuracy of the estimation. Firstly, it is necessary to calculate the predicted value of the system at the moment of k according to the input value of the system and the optimal value of the system state variable at the moment of k−1 . The specific calculation method is shown in Eq. 5.


(5) $$X_{k|k-1}=F_{k}X_{k-1|k-1}+B_{k}u_{k}$$

Eq. 5 describes the current result state $X_{k|k-1}$ predicted at the moment of k . The optimal value at the moment of k−1 is represented by $X_{k-1|k-1}$ . $F_{k}$ denotes the transformation matrix in the state of $X_{k-1|k-1}$ , which serves as the foundation for the algorithm’s predictions on state variables. Furthermore, $B_{k}$ denotes the transformation matrix acting on the control variables and $u_{k}$ indicates the control gain of the current state. The system covariance at k is predicted using the system covariance at k−1 , as calculated in Eq. 6.


(6) $$P_{k|k-1}=F_{k}P_{k-1|k-1}F_{k}^{T}+Q_{k}$$

Eq. 6 specifies the calculation of the Kalman gain, where $P_{k|k-1}$ represents the system’s covariance matrix at time k , $P_{k-1|k-1}$ represents the covariance matrix of the system at time k−1 , and $Q_{k}$ represents the covariance of the system’s process noise. By recursively using the prediction step of Eq. 6, the system covariance at time *k* can be obtained. The predicted value of the covariance matrix at time k is used to calculate the Kalman gain, as shown in Eq. 7.


(7) $$K_{g}=\frac{P_{k|k-1}H_{k}^{T}}{H_{k}P_{k|k-1}H_{k}^{T}+R_{k}}$$

In Eq. 7, $K_{g}$ is the Kalman gain, the Kalman gain is a key parameter in the Kalman filter, which is used to adjust the trade-off between measured values and system model predictions, in order to estimate the optimal value of the state variable. When the Kalman gain is large, the system trusts the measured values more and has less weight on the prior estimates. This means that the system is more sensitive to changes in measured values and updates the estimated values of state variables with the measured values as the dominant factor. This usually produces better results when the measurement values are more accurate and the noise is smaller. When the Kalman gain is small, the system has a higher weight on the prior estimate and is relatively less confident in the measured value. This means that the system is relatively conservative and tends to trust the predictions of the system model. This can provide more stable estimation results in situations where measurement values are less accurate and noise is high. So, by adjusting the size of the Kalman gain according to the actual situation, more accurate and stable state estimates can be obtained under various measurement and noise conditions. $H_{k}$ is the object prediction matrix; $R_{k}$ is the covariance matrix of the object measurement noise. The optimal values of the state variables at moment k are then calculated as shown in Eq. 8.


(8) $$X_{k|k}=X_{k|k-1}+K_{k}\left(Z_{k}-H_{k}X_{k|k-1}\right)$$

In Eq. 8 $X_{k|k}$ is the optimal estimated value of the state variable at moment k ; $Z_{k}$ is the measured value of the object. The purpose of this equation is to obtain the best estimate of the system state based on current observation data and past state estimation through the estimation process of the Kalman filtering algorithm. This best estimate can be used to restore real data, that is, to describe or reconstruct the actual state of the system through the estimated value. Finally, the covariance matrix at moment k is derived based on the above results, which is shown in Eq. 9.


(9) $$P_{k|k}=\left(I-K_{k}H_{k}\right)P_{k|k-1}$$

In Eq. 9, $P_{k|k}$ is the covariance matrix of k moment; I is the unit matrix. After finding out the covariance matrix at the moment of k , the Kalman output value at the moment of k+1 can be introduced. In the Kalman filtering algorithm, the covariance matrix at time k is derived to describe the accuracy and credibility of state estimation at time *k*. The relationship between the Kalman filtering algorithm and the previous equation lies in the recursive fusion of previous estimates and observations to obtain the optimal state estimate and covariance matrix at the current time. By continuously predicting and updating cycles, the Kalman filtering algorithm can continuously correct and update state estimates, making the estimation results closer to real data.

### 3.2 Design of Tensorflow-based waste recycling management system

**Figure 5:**
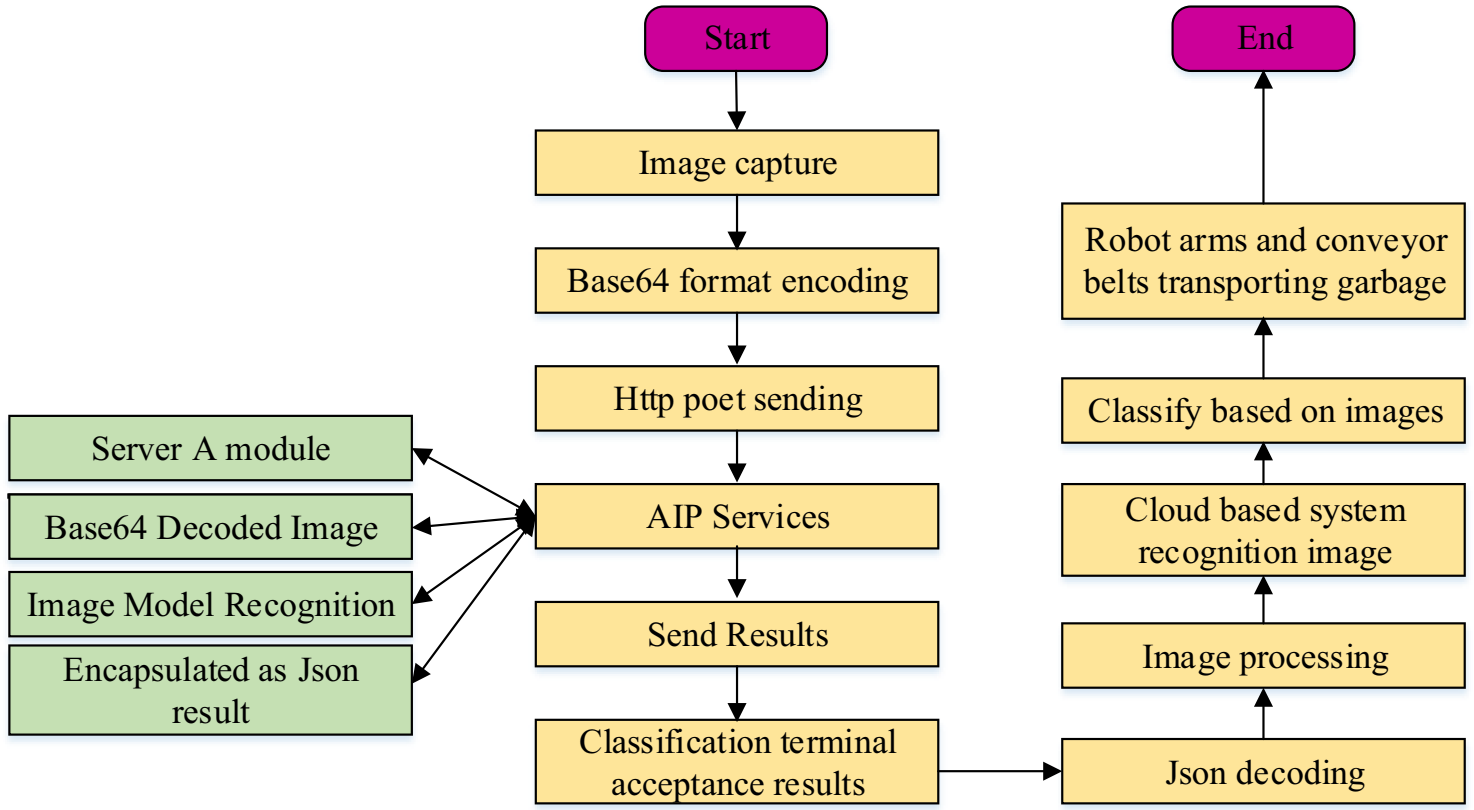
Flowchart of the operation of the Tensorflow-based rubbish collection management system.

With the acceleration of urbanization, waste disposal has become an urgent problem. In this study, a Tensorflow-based waste recycling management system is designed for some common recyclable materials (e.g., paper, plastic, metal). The specific flow of the operation of the recycling system is shown in Figure 5.

**Figure 6:**
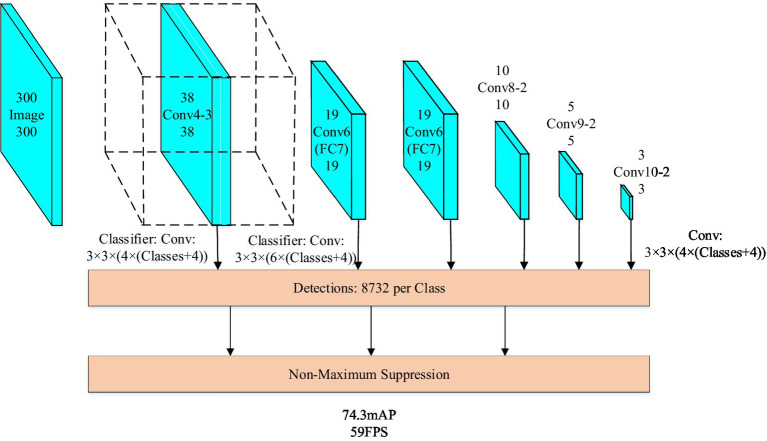
Flowchart for SSD model training.

As shown in Figure 5, the recycled rubbish is first dynamically photographed, and then the photographed image of each rubbish is converted into Base64 form of encoding and transmitted to the API server through the HttP Post transmission protocol. Afterwards, the image data is decoded by the API server, and then the rubbish subject is recognized by an image algorithm, and at the same time the recognition results are transmitted to an image processing unit. Finally, the processing results are uploaded to a cloud-based system, which carries out classification and identification and then commands the robotic arm and conveyor belt to sort and transport the waste (27–29). In this system, the core processing unit is the image processing unit. The image processing unit of this system is based on the TensorFlow algorithm, which mainly preprocesses the captured rubbish images through OpenCV, and then uses the SSD model training to classify the target images in a directional way. In the system, after preprocessing and annotating garbage images, they are input into the SSD model. The SSD model will locate the garbage in the image, determine its location and bounding box, and classify the garbage. Through such positioning and classification, the system can accurately segment garbage items from images and obtain their category information. In addition, the SD model uses deep learning algorithms, which can be trained on large-scale datasets to learn rich feature expression and pattern recognition capabilities, enabling the SSD model to effectively classify different types of garbage. The SSD model training process is shown in Figure 6.

For images of recycled rubbish, the dot product needs to be calculated over the entire image, as shown in Eq. 10.


(10) $$f\left(x\right)=W^{T}x+b$$

In Eq. 10 W is the filter and x = input image b = deviation. At the end of each convolutional layer, SDD obtains the activation map of the output image and the activation function is shown in Eq. 11.


(11) $$F\left(x\right)=\max\left(0,x\right)$$

The minimum value domain of this activation function is set to 0. After that the Otsu algorithm is used for thresholding to isolate it from the background. The Otsu algorithm uses a threshold value to classify the pixels into two classes, which is calculated as shown in Eq. 12.


(12) $$\delta_{\mathrm{intra}}^{2}\left(T\right)=n_{B}\left(T\right)•\delta_{B}^{2}\left(T\right)+n\left(T\right)•\delta_{o}^{2}\left(T\right)$$

In Eq. 12 $n_{B}$ and $n_{o}$ are the number of pixels below or above the threshold; $\delta_{B}$ and $\delta_{o}$ are the variance of $n_{B}$ and $n_{o}$ ; and T is the selected threshold. The image is then subjected to anti-disturbance chemistries and transformed into a Gaussian filtered convolutional image by filtering and binarization. A typical Gaussian function equation is shown in Eq. 13.


(13) $$f\left(x\right)=a\exp\left[\frac{-{\left(x-m\right)}^{2}}{2\sigma^{2}}\right]$$

In Eq. 13, a is the constant; m is the mean; σ is the standard deviation. In image processing, Gaussian filtering is commonly used to smooth images and reduce noise. The integral equation can be used to describe the smoothing transformation of pixel values in images by Gaussian filtering. If the image with *N* × *N* pixels is $f\left(x,y\right)$ , then the description method for the pixel value at any point in the image is shown in Eq. 14.


(14) $$f\left(x,y\right)=\int \int f\left(u,v\right)\exp\left[-{\left\{u-x\right\}}^{2}+\frac{{\left(v-y\right)}^{2}}{2\sigma^{2}}\right]\mathrm{dudv}$$

In Eq. 14 $f\left(u,v\right)$ is the pixel value of point $\left(u,v\right)$ on the original image; σ is the Gaussian filtering parameter. Gaussian functions can convolve images to reduce noise and details, making the image smoother. Secondly, by applying a Gaussian filter to the image, the edges and details in the image can be emphasized, making it clearer and easier to analyze. Again, Gaussian functions can be used to generate various types of noise, thereby helping to develop and test the robustness and performance of image processing algorithms. The image is edgewise detected after noise reduction and then passed to the contour map. Firstly, edge detection can help determine the contour of an object, thereby obtaining its precise shape for better subsequent processing. Secondly, edge detection can use edges as features for classification and matching in subsequent object recognition. Thirdly, edge detection can provide image contour information, which is helpful for image enhancement and denoising, and provides a foundation for subsequent image enhancement steps. The contour localization classification learning is performed by the SSD model to determine the recyclable rubbish classification information, in addition, the SSD model is trained to classify the rubbish image through the cross-validation method to improve the image recognition rate (30, 31). Cross validation is a universal machine learning method that is one of the commonly used methods for evaluating model performance. When training SSD models for garbage image classification, cross validation can first detect whether the model has overfitting problems. By dividing the training data into several parts, only one part is used for training and the other parts are used for validation in each training session. This avoids the model from overly relying on specific training parts Data can effectively prevent overfitting problems from occurring. Secondly, the model can be trained and validated multiple times to obtain multiple sets of results, thereby better evaluating the performance of the model. Thirdly, through multiple training and validation, more comprehensive and objective model performance data can be obtained, thereby better evaluating the robustness of the model. After the processing of garbage images is completed, the garbage collection system will automatically perform corresponding operations based on the results of image processing and classification to efficiently collect and process garbage. Firstly, based on the garbage classification results, the robotic arm can grab and place it in the corresponding container or area for classification and treatment. Then the conveyor belt can transport the garbage to the designated location based on the classification information of the garbage. Finally, if the garbage collection system detects abnormal situations, such as garbage classification errors or garbage that cannot be processed, it can trigger corresponding alarms and exception handling measures. In addition, all data is recorded and saved by the garbage collection system.

### 3.3 Intelligent traffic management system design

**Figure 7:**
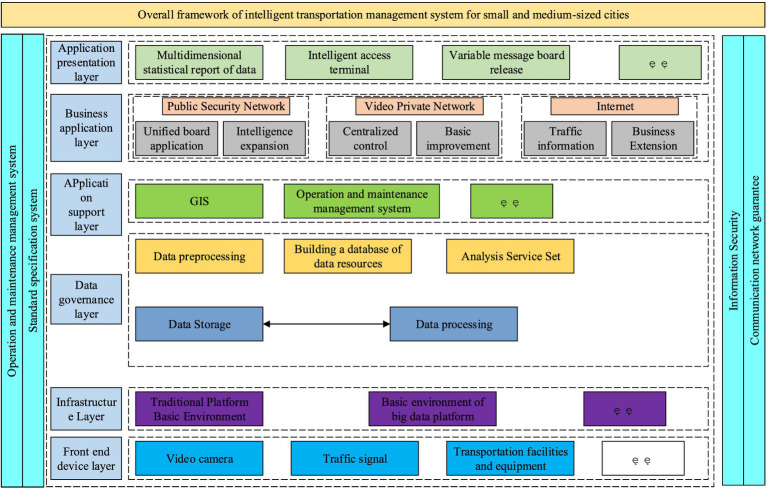
Overall architecture of urban intelligent traffic management system.

With the rapid development of China’s economy, the number of families with vehicles is increasing day by day, and the city is facing great traffic pressure. For this reason, an intelligent traffic management system is designed to alleviate the traffic pressure in the city, and the general framework of the system is shown in Figure 7.

**Figure 8:**
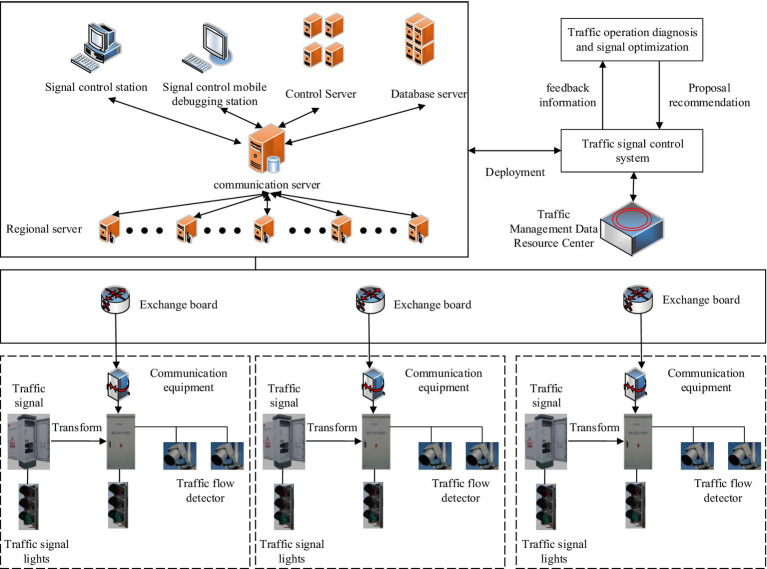
Physical structure of urban traffic signal control system.

Figure 7 presents the traffic management system, which comprises six main layers. The application presentation layer emphasizes the decision-making aspect of data support, provides visual display of traffic data, and enables data access. The business application layer depends on various networks including the public security network, video network, and Internet to accomplish urban traffic monitoring and management. In the application support layer, a geographic information support system and an operation and maintenance management system are developed to enhance the integrated management of traffic equipment and facilities data and other resources. In the data governance layer, it primarily pre-processes traffic data and establishes a data resource library to accomplish the analysis and storage of traffic data. In the infrastructure layer, the primary focus is on the sustained coexistence of big data and traditional architecture. In the front-end equipment layer, the primary objectives are to test the construction of key nodes and manage both new and old equipment of the traffic system. Intelligent traffic management does not need to be completely new, but can be expanded according to the infrastructure of the combined city. It can be carried out mainly in three aspects, namely, upgrading business application functions, improving the basic support system and upgrading the level of traffic signal intelligence. The first is the enhancement of business application functions. The study can make use of the data and analysis results provided by the intelligent traffic system to achieve dynamic traffic flow scheduling and optimization. Through intelligent signal control, signal timing can be adjusted according to real-time traffic conditions to reduce congestion and improve road efficiency. Accurate navigation and route planning functions are also provided through ITS to help drivers choose the best routes and provide re-planning suggestions based on real-time traffic conditions to avoid congested road sections and shorten travelling time. Vehicle monitoring equipment and technology provided by ITS can also be used to achieve the identification and recording of traffic offences, assisting traffic management authorities to carry out accurate law enforcement. At the same time, it can also provide real-time vehicle flow monitoring information to optimize vehicle traffic planning. The second is to improve the basic support system, for which it is sufficient to improve from the following three points. 1. Provide unified map service and development interface for ITS to realize the visual display of multi-dimensional traffic elements, multi-dimensional traffic management information and multi-layer business data on the map. 2. Strengthen the data resource management and sharing of the existing traffic management system, and construct a unified and standardized data resource pool. 3. Improve the information security level protection, establish a security guarantee system, improve the security boundary equipment of the public security network, the special video network and the Internet, and ensure reliable and efficient cross-network data exchange. The third is to improve the level of traffic signal intelligence, which is the main method to solve the problem of urban traffic congestion and one of the basic applications of intelligent traffic management system. For this reason, the urban signal control system architecture is designed, which is shown in Figure 8.

In Figure 8, the signal control system is an intelligent traffic management system that uses signal self-harvested traffic flow data and road condition data shared with Internet mapping enterprises to evaluate and diagnose intersection operation and generate optimized control schemes for selection and issuance by the signal control system. The system not only has the functions of conventional parameter configuration and online control scheme configuration, but also can carry out regional vehicle route co-ordination and traffic arterial vehicle route co-ordination, and at the same time supports the functions of signal priority for special duty vehicles and signal priority for public transport vehicles. In addition, the system can control and optimize the traffic flow in tidal and oversaturated lanes, thus improving the efficiency of road access.

## 4 Performance testing and simulation of the system simulation experiments

This chapter focuses on verifying the effectiveness of the three systems designed and analyzing the results. For the Living Environment Management MS, the experiment tested various parameters of residential water quality, urban air and noise level and compared them with the real values; for the Garbage Recycling Management System, the experiment tested the accuracy of the system in classifying four types of rubbish: paper, metal, plastic, and glass; and for the Intelligent Traffic Management System, the experiment monitored the congestion of the city’s traffic at various times of the day over a period of 30 days.

### 4.1 Living environment inspection management system simulation and inspection experiment

**Figure 9:**
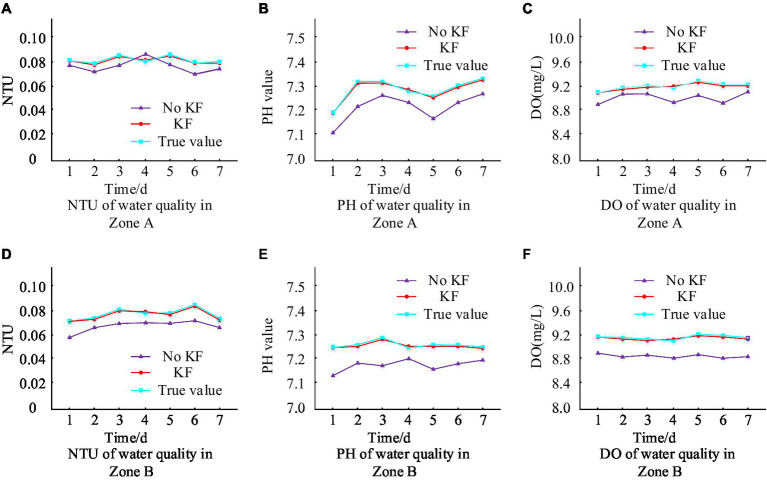
Comparison of water quality test results.

To verify the accuracy of the MS of urban living environment designed by the Institute, A district in the south and B district in the north of a city are selected as experimental sites for dividing each area into different collection points, and conduct a 7 days data collection and detection through the collection points. The experimental samples to be tested are taken in A and B districts, and then examined in the laboratory with professional testing instruments, and the test results are taken as the real values. Use SPSS software to process the data collected from areas A and B every day, first perform data cleaning and filtering to remove invalid and abnormal data, and ensure the accuracy and reliability of the sample data. Then, the true values found in the experiment are compared with the data of the detection system designed by the research institute, and the error between them is calculated to verify the detection accuracy of the system designed by the research institute, and the specific results are shown in Figure 9.

**Figure 10:**
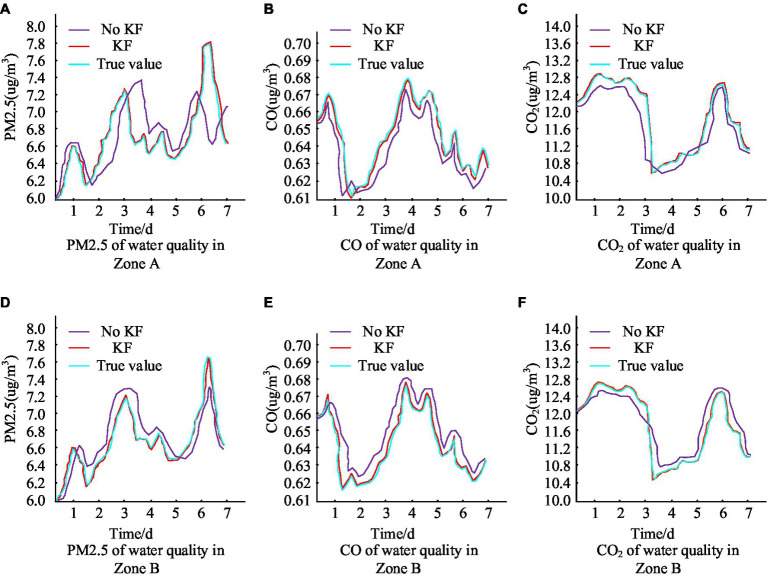
Comparison of urban air ambient test results.

Figures 9A,D show the results of water turbidity testing in two areas of the city, A and B, respectively. Turbidity detection can quickly evaluate the clarity of water quality and intuitively reflect the content of impurities in the water. High turbidity may indicate the presence of pollutants in the water, affecting its visibility and drinking safety. Water turbidity in area A within a week of the real value of the change range of 0.076–0.086, the water turbidity in area B within a week of the real value of the change range of 0.071–0.085, the two areas have a slight difference, which may be due to the rainfall and other natural factors The slight difference between the two zones may be caused by impurities brought about by rainfall and other natural factors. Observing the trend of the curve in the figure, the turbidity detection value of the MS with KF algorithm is almost the same as the real value, and the overall relative error is not more than 1.0%. However, the MS without KF algorithm has a relatively large difference with the real value, and the relative error of water quality turbidity detection is at least more than 10%. Figures 9B,E indicate the dynamic change of water quality PH values in areas A and B of the city. PH value is an indicator of the acidity and alkalinity of water, which affects the taste, solubility, and impact on organisms of water. The normal pH value of drinking water should be between 6.5 and 8.5. Excessive or insufficient pH values may lead to abnormal acidity and alkalinity of the water, affecting human health. The application of KF algorithm processing improves the tracking of the real value change process in both areas A and B. Furthermore, the maximum relative error between the two is not exceeding 1.4%. Figures 9C,F display the outcomes of identifying dissolved oxygen values in the water quality of A and B areas of the city. Testing the dissolved oxygen rate can help evaluate the health level of water bodies and take timely measures to maintain the oxygen balance of water bodies. It is observable from the curve trends that the KF algorithm has brought the system closer to the actual measurement, reducing maximum relative errors to 1.2% or less. This was followed by the detection of PM2.5 and CO/CO2 concentrations in the urban air environment, which is presented in detail in Figure 10.

Figures 10A,D show the results of PM2.5 detection in the air of city A and B. It can be seen that the PM2.5 fluctuation range of the air in the city in the recent week is 6.0 μg/m^3^–7.8 μg/m^3^. Observing the fluctuation curves of the real value of PM2.5 and the detected value of the system with the addition of the KF algorithm, it can be seen that the two values almost overlap, and the maximum relative error is not more than 1.5 per cent. On the other hand, the fluctuation curve of the PM2.5 system without KF algorithm has a larger gap with the real value. Figures 10B,E show the results of air CO concentration detection in city A and B, respectively, and it can be seen that the fluctuation range of CO concentration in the city in a week is 0.61 μg/m^3^–0.68 μg/m^3^. Similarly, according to the trend of the three curves, it can be seen that the CO concentration values processed by the addition of KF algorithm better reflect the real situation of the detected values, while the CO concentration values without the addition of KF algorithm detected values are then more different from the real values. Figures 10C,F show the results of air CO2 concentration in city A and B, respectively, and the CO2 concentration fluctuates in the city in a week in the range of 10.40 μg/m^3^–12.84 μg/m^3^. Observing the test curves of the two graphs, it can be seen that the detected values of the KF algorithm are close to the real values. Then the noise level of the city was detected in three time points every day with city A as the test object, and the test results are shown in Table 1.

**Table 1:** Statistics of noise level detection during one week in city A area.

/	/	True value (dB)	KF system detection value (dB)	Detected value without KF system (dB)	Absolute error 1	Absolute error 2
1	8:00	35.6	35.4	39.1	0.0056	0.0983
	14:00	42.6	41.3	36.9	0.0305	0.1338
	18:00	49.6	48.4	41.6	0.0241	0.1612
2	8:00	33.6	32.6	41.6	0.0297	0.2380
	14:00	45.9	44.3	40.3	0.0348	0.1220
	18:00	50.9	51.3	42.9	0.0078	0.1571
3	8:00	33.9	32.8	37.6	0.0324	0.1091
	14:00	45.6	44.3	38.6	0.0285	0.1535
	18:00	52.6	51.9	44.2	0.0133	0.1596
4	8:00	37.9	36.5	30.8	0.0369	0.1873
	14:00	48.5	47.1	39.4	0.0288	0.1876
	18:00	56.3	55.4	49.6	0.0159	0.1190
5	8:00	37.2	35.6	46.2	0.0430	0.2419
	14:00	42.5	44.3	44.3	0.0423	0.0423
	18:00	53.6	55.9	42.8	0.0429	0.2014
6	8:00	36.5	34.8	41.9	0.0465	0.1479
	14:00	45.8	44.8	54.1	0.0218	0.1812
	18:00	43.2	44.5	52.3	0.0300	0.2106
7	8:00	36.8	35.8	45.2	0.0143	0.2282
	14:00	45.9	44.5	51.3	0.0305	0.1176
	18:00	58.3	56.3	41.6	0.0343	0.2864

Absolute error 1 refers to the absolute error between the detected value of the system with KF added and the true; absolute error 2 refers to the absolute error between the detected value of the system without KF added and the true.

From Table 1, the noise level of the city is considered good. The difference between the system value of the noise level with KF algorithm and the real detection value is small, and the maximum relative error is 0.0465, while the difference between the system value of the noise level without KF algorithm and the real detection value is large, and the maximum relative error is as high as 0.2864. Therefore, the above data shows that the system designed by the institute can stably achieve the real-time monitoring of the data of the city’s environment, and the accuracy of the detection is more than 95%. 95% or more.

### 4.2 Simulation and testing experiment of garbage collection management system

**Figure 11:**
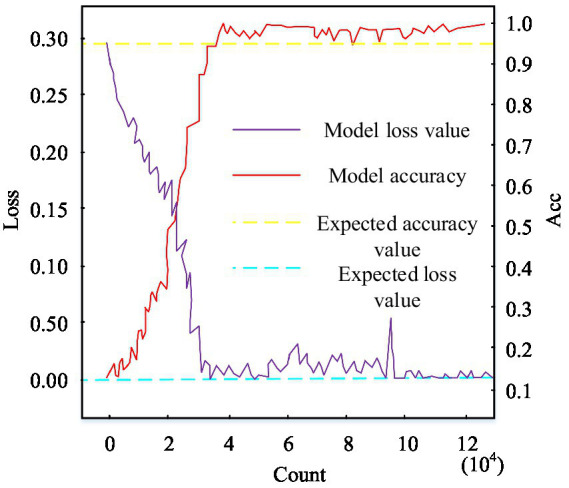
Training model loss-ACC plot.

In order to verify the effectiveness of the designed waste recycling management system for sorting and processing, the waste sorting test in the city is carried out on Debian system using Go language and SpotGarbage waste identification dataset. The main objects of this waste classification and recycling are paper, metal, plastic and glass. The results of the previous test are: the correct rate of paper classification is 63.9%, the correct rate of metal classification is 76.3%, the correct rate of plastic classification is 63.5%, and the correct rate of glass classification is 68.9%. The classification error at this point can be seen to be large, so the model needs to be trained. The feasibility of model training is tested first, and the training data set is transported to the SSD model, which is used to correspond the pictures to the labels of the classified objects, and the detection results are shown in Figure 11.

**Figure 12:**
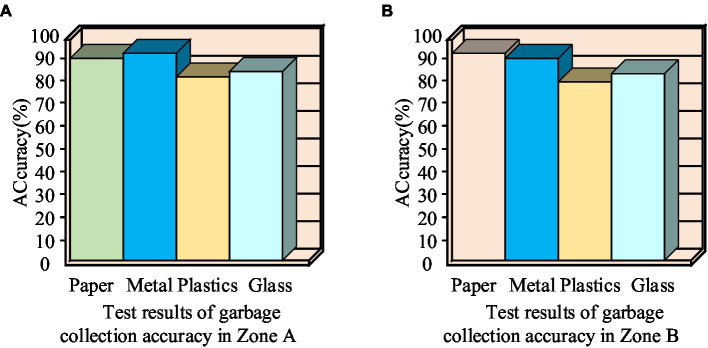
Municipal waste sorting and recycling accuracy test results.

In Figure 11, with the increase of the number of training steps, the SSD model accuracy rises sharply, while the SSD model loss value decreases sharply. When the number of training steps is about 40,000 steps, the SSD model accuracy is gradually stable and finally converges to 100%, which meets the desired value. When the number of training steps is about 31,567 steps, the SSD model loss value gradually stabilizes and eventually converges to 0%, satisfying the expectation value. Therefore, as the number of training times increases, the training effect of the SSD model gradually enhances, which meets the requirements of the classification and recycling system and verifies the feasibility of the SSD classification model. As for the problem of low classification accuracy of the system due to insufficient model training, crawler software is used to search for pictures on the web to increase the training dataset. Then the test is carried out in the rubbish collection sites in city A and B. The results are shown in Figure 12.

As can be seen in Figure 12, at the waste recycling test site in urban area A, the accuracy of sorting and recycling of four types of waste: paper, metal, plastic and glass were 92.1, 95.3, 85.6, and 88.9%, respectively. In City B, the accuracy rates for paper, metal, plastic and glass were 93.4, 92.3, 83.6, and 87.6%, respectively. Plastic waste can be identified as having a poorer sorting and recycling accuracy rate, which may be due to the fact that different types of waste have different characteristics and forms. Wastes such as paper, metal, plastic and glass differ significantly in appearance, material and texture. These differences can affect how accurately a machine learning model can categorize and identify different wastes. For example, paper usually has a smooth surface, metal may have a shiny appearance, plastic has an irregular shape, and glass is transparent and fragile. Therefore, more training data on plastic waste is needed as a way to improve the generalization ability of the classification model and hence the classification accuracy.

### 4.3 Intelligent traffic management system simulation and testing experiment

**Figure 13:**
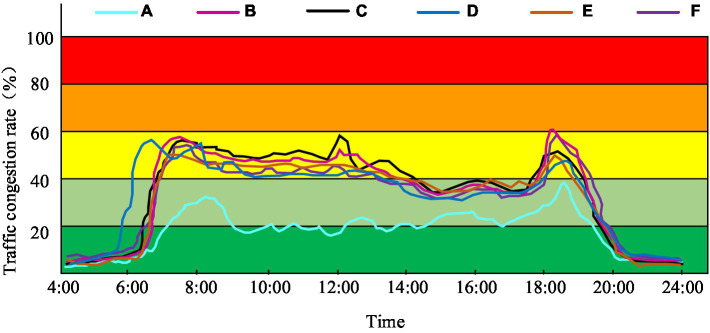
Results of monitoring traffic congestion in six areas of the city over the course of a day.

In order to verify the effectiveness of the improved urban intelligent traffic management system, six regions of a city with similar traffic conditions, A, B, C, D, E, and F, are selected for the study. The traffic management system in area A is improved, while the other five areas are used as controls. The traffic sections in each area are detected in real time by the monitoring system, and if the vehicle stopping time of a section is more than 3 min, it was determined that the section was congested. The six study areas of the city were monitored for a period of 30 days, and Google Maps was used to count the results, which were then synthesized into traffic congestion at various times of the day, as shown in Figure 13.

During the non-congested hours (e.g., 0:00–6:00, 20:00–24:00), congestion rates in the six zones exhibit minimal differences, ranging from approximately 3 to 8%, as seen in Figure 13. However, after 6:00 pm, the city’s congestion rate begins to increase rapidly, albeit in Area A, where ITSM measures are adopted, it shows a slower increase. Notably, this area experiences two congestion peaks, one around 8:20, where the traffic congestion rate is around 34%, and the other around 18:40, recorded at approximately 38%. Traffic congestion is consistent across the other five areas, with rates increasing from 6:00 a.m. and sustaining above 40%. During evening rush hour, the rate of traffic congestion in areas where intelligent traffic management measures have been implemented can peak at 60%. Overall, data from all hours of the day indicates that the adoption of such measures reduces the congestion rate of urban traffic by a minimum of 20%. Consequently, the implementation of intelligent traffic management measures can lead to a reduction in vehicle emissions. This is because during periods of congestion, vehicles’ engines tend to idle or operate at low speeds. In turn, this inefficiency results in a decreased combustion efficiency of the vehicle, thereby increasing tailpipe emissions. Reduced traffic congestion enables vehicles to travel smoothly, resulting in decreased idling and low-speed travel, consequently reducing fuel wastage and exhaust emissions. Furthermore, the time required for stopping and starting of vehicles is lowered, thus reducing fuel consumption and tailpipe emissions. Adoption of ITSM measures is anticipated to decrease tailpipe emissions by a minimum of 20 t per year in six sections of the city.

## 5 Conclusion

To achieve a comprehensive improvement of SCESMPH, the study devised three improvement systems. For the detection of living environment issues faced by residents of smart cities (SC), this study have designed the living environment data management system (MS). This system comprehensively utilizes sensor technology to collect data parameters for urban air quality, noise, and water quality. The data is then sent to the processor via internet technology, where the KF algorithm is used for precise data processing. Finally, the detection data is presented on the client side. Conduct a 7 days data monitoring of A and B districts in a certain city, and use the ThingWorx software developed by PTC company to analyze and organize the collected data. The study revealed that the incorporation of the KF algorithm led to a maximum detection error of water quality parameters not exceeding 1.4%, air quality parameters not exceeding 1.2%, and a noise level not exceeding 4.56%. While the MS of living environment data can enhance the precision of data analysis after employing the KF algorithm, there still remains a certain degree of inaccuracy. Further progress can be made in optimizing the algorithm and sensor technology. Future studies will conduct more comprehensive research for heightened accuracy in environmental detection and automated management.

To enhance the cleanliness of the SC environment, a waste recycling system has been developed. This system is built on the Tensorflow framework and uses the SSD model to improve the accuracy of identifying the waste images. The system then commands the robotic arm to classify and transport the waste based on the classification images. Conduct garbage classification testing for cities using Go language and SpotGarbage garbage recognition dataset on the Debian system. The findings indicate that the system attains a maximum accuracy rate of 93.4, 95.3, 85.6, and 88.9% for four categories of waste, specifically paper, metal, plastic, and glass, in accordance with the standards of waste classification and recycling. Whilst the waste recycling management system exhibits great efficiency in sorting and recycling paper, metal, plastic, and glass, the system’s recognition accuracy for other waste types necessitates improvement.

To enhance the wellbeing of South Carolina inhabitants and decrease metropolitan contamination, an intelligent traffic management system has been devised. The aforementioned scheme showcases a multifaceted architecture and can carry out several tasks, including enhancing routes, amplifying vehicular communication whilst offering swift police responses. Conduct a 30 days road condition monitoring of six research areas in the city, and use Google Maps to collect monitoring results, which are then comprehensively compiled into traffic congestion levels at different time periods within a day. The results indicate that, in the district where this traffic management system is implemented, there is a minimum 20% reduction in urban traffic congestion. Whilst it is true that the intelligent traffic management system can successfully alleviate traffic congestion, it is important to note that this comes with a few technical and managerial obstacles involved with its practical implementation. These hindrances require further refinement and improvement.

## Data availability statement

The original contributions presented in the study are included in the article/supplementary material, further inquiries can be directed to the corresponding authors.

## Author contributions

BL: Investigation, Writing – original draft. JG: Investigation, Writing – original draft. CW: Investigation, Writing – review & editing.
